# Supplementary material for: A UMLS-based spell checker for natural language processing in vaccine safety
Source: BMC Med Inform Decis Mak. 2007 Feb 12;7:3. doi: 10.1186/1472-6947-7-3 (PMC1805499; doi:10.1186/1472-6947-7-3)
Supplement: Additional file 13 — RAP application directory. Sets up directory for RAP (RDF application for PHP) source code [file 1472-6947-7-3-S13.gz › rap/doc/tests/test_ManipulateDbModel.php.txt]

Test Store Models in Database

php
// Include RAP
define("RDFAPI\_INCLUDE\_DIR", "./../api/");
include(RDFAPI\_INCLUDE\_DIR . "RDFAPI.php");
## 1. Connect to MsAccess database (via ODBC)
## ------------------------------------------
// Connect to MsAccess (rdf\_db DSN) database using connection settings
// defined in constants.php :
$rdf\_database = ModelFactory::getDbStore();
## 2. Load a DbModel
## -----------------
$dbModel = $rdf\_database-getModel("example1.rdf");
// Output the model as HTML table
$dbModel->writeAsHtmlTable();
echo "  
  
";
## 3. Add a statement tho the DbModel
## ----------------------------------
// Ceate a new statement
$statement = new Statement(new Resource("http://www.w3.org/Home/Lassila"),
new Resource("http://description.org/schema/Description"),
new Literal("Lassilas persönliche Homepage", "de"));
// Add the statement to the DbModel
$dbModel->add($statement);
// Output the string serialization of the DbModel
echo $dbModel->toStringIncludingTriples();
echo "  
  
";
## 4. Search statements
## ---------------------
// Search for statements having object $literal
$literal = new Literal("Lassilas persönliche Homepage", "de");
$res = $dbModel->find(NULL, NULL, $literal);
// Output the result
$res->writeAsHtmlTable();
echo "  
";
## 5. 5. Replace nodes and serialize the DbModel to XML/RDF
## --------------------------------------------------------
// replace a literal
$dbModel->replace(NULL, NULL, new Literal("Lassilas persönliche Homepage", "de"),
new Literal ("Lassila's personal Homepage", "en"));
// Serialize to RDF
$dbModel->writeAsHtml();
echo "  
  
";
## 6. Remove a statement
## ---------------------
$dbModel->remove(new Statement (new Resource("http://www.w3.org/Home/Lassila"),
new Resource("http://description.org/schema/Description"),
new Literal ("Lassila's personal Homepage", "en")));
// Output the DbModel
$dbModel->writeAsHtmlTable();
echo "  
";
## 7. Generate a MemModel and compare both models
## ----------------------------------------------
// Generate a MemModel
$memModel = $dbModel->getMemModel();
// Compare this DbModel withe the generated MemModel
$res = $dbModel->equals($memModel);
if ($res)
echo "models are equal";
else
echo "models are different";
echo "  
";
## 8. Save DbModel to file
## ----------------------------------------------
// Save DbModel to file (XML/RDF)
$dbModel->saveAs("Output.rdf");
// Save DbModel to file (N3)
$dbModel->saveAs("Output.n3");
// close the DbModel
$dbModel->close();
?>
